# Action inhibition in a sport-specific paradigm: examining the limits of action control in basketball

**DOI:** 10.1007/s00426-024-02010-2

**Published:** 2024-08-03

**Authors:** Carolin Wickemeyer, Iris Güldenpenning, Matthias Weigelt

**Affiliations:** Department of Sport & Health, Paderborn University, Warburger Straße 100, 33098 Paderborn, North Rhine-Westphalia Germany

## Abstract

To investigate the point where inappropriate defensive movements can no longer be inhibited and to validate suitable stimulus material for constructing a basketball-specific anticipation-response-inhibition task, two experiments were conducted. In Experiment 1, participants without basketball expertise (N = 25) watched a video of a basketball jump shot and were asked to release the space bar at the point when the ball leaves the player's fingertips (go-trials). In 25% of all trials, the video was stopped prematurely and participants should withhold their finger-lift response (stop-trials). A staircase-tracking algorithm was used to adjust the point-in-time when the jump shot was stopped in a way that participants’ inhibition rate was at 50% (reflecting the so called “point-of-no-return”, PNR). In Experiment 2, the stimulus material was adapted so that stop-trials simulated a pump fake. The PNR in Experiment 1 was located 187 ms and in Experiment 2 177 ms before the point of ball release. Precision performance benefit from practice across blocks and participants delayed their responses after stop-trials in a subsequent go-trial, which reflects strategic post-stop-trial adjustments. Based on the comparable results of previous studies, the given stimulus material is suitable for investigating response inhibition skills in dynamic sport-specific environments.

## Introduction

**Fig. 1:**
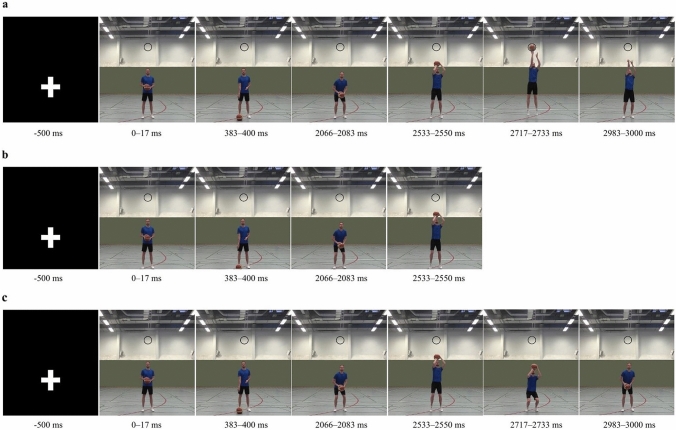
Displayed are still frames taken as examples from the video stimulus. Note: **a** Basketball jump shot used in the go-trials. **b** Basketball pump fake for the stop-trials in Experiment 1. **c** Modification of the stimulus material for the stop-trials in Experiment 2. In **c**, the video stopped and reversed 2533 ms after the video start, which is equivalent to the video frame that displays the PNR

In many team sports, it is a common practice to perform deceptive actions to trick the opponent, which provides the attacker with additional time to pursue the intended action goal. In basketball, for example, pump fakes are used to get the defensive player out of position (Bösing et al., 2019). According to the taxonomy of Weigelt and Güldenpenning (2022), the pump fake is a deception in which the attacker provides action-irrelevant (mis-)information by only pretending to throw (i.e., pump fake) in the first part of the action, before stopping the movement, followed by another (real) throwing action (i.e., jump shot) in the second part. Thus, the pump fake in basketball is a combination of two successive actions (cf. Figure 1, Weigelt & Güldenpenning, 2022). Its effectiveness is based on the reaction of the defensive player to the fake throw in the first part of the action. Assuming that the attacker will throw the ball to score with a jump shot, the defender reaches up and initiates a blocking action. At a certain point in time, this blocking action to the fake attempt cannot be inhibited anymore by the defender, putting him/her out of position to block the (real) throw in the second part. In order to successfully defend the attacker, the defender must anticipate the attacker’s intention. Such anticipation skills are not only crucial for the success in basketball but also in many other game sports (Güldenpenning et al., 2017; Williams & Jackson, 2019). In this regard, kinematic (e.g., movement speed, extent, and trajectory) and contextual (e.g., game score, field position, probability of action outcome) sources of information are exploited during the social interaction of two or more players (Loffing & Canal-Bruland, 2017). As previous research has shown, expert athletes are better able to use these external sources of information and therefore, possess better anticipation skills than novices (e.g., Loffing & Canal-Bruland, 2017; Müller & Abernethy, 2012; Williams & Jackson, 2019). These better anticipation skills are based on more experienced visual search behavior and the superior ability to pick up perceptual and contextual cues (Mann et al., 2007; Loffing & Canal-Bruland, 2017; Klostermann et al., 2018). Evidence for such expert superiority effects has been provided for several team sports, like volleyball (Güldenpenning et al., 2013), rugby (Gabbett & Abernethy, 2013; Jackson et al., 2006; Mori & Shimada, 2013), soccer (Savelsbergh et al., 2002; Smeeton & Williams, 2012; Williams, 2000), and team handball (Cañal-Bruland & Schmidt, 2009; Cañal-Bruland et al., 2010).

In basketball, the kind of sport examined in the present two experiments, differences between expert and novice performance were observed for anticipating free shots (Aglioti et al., 2008), processing head fakes (Güldenpenning et al., 2017; Weigelt et al., 2017), judging pass fakes (Sebanz & Shiffar, 2009), anticipating shot fakes (Meyer et al., 2022c), and for a compound receiving, dribbling, and shooting action sequence (Meyer et al., 2022b). Given the superior anticipation skills of experts in basketball (and in many other team sports), the most recent observation reported by Meyer et al. (2022a) is most surprising: When the authors examined shot fakes during real games in the National Basketball Association (NBA), 73% of all shot fakes were successful and increased the likelihood of offensive scoring. Obviously, even outstanding anticipatory skills do not prevent experts from falling for fakes. The reason for this may be, that, even if a defensive player recognizes the deception of the attacker after some point in time during its execution, stopping the prepared but no longer relevant response (i.e., the blocking action) is not possible anymore. Instead, once this point in time is surpassed, the blocking action cannot be inhibited and will be executed, inevitably. This provides the attacker with extra time to score. This could be related to the fact that a defensive action must be planned and initiated at an early stage in order to intercept a throw in time, however, the defensive action must be inhibited if the attacker performs a pump fake. This raises the question up to which point in time an inappropriate defensive movement can successfully be inhibited.

This situation in basketball is reminiscent of what has been referred to as the point-of-no-return (PNR) in the research on response inhibition in experimental psychology (Logan, 2015; Osman et al., 1986). The PNR denotes the 50% probability of successful and unsuccessful response inhibition (Barlett, 1958, as cited in Logan, 2015) and can be determined experimentally either with the stop-signal task (SST; Lapping & Erikson, 1966; for reviews, see Verbruggen & Logan, 2008a) or the anticipation-response-inhibition (ARI) task (e.g., Coxon et al., 2007; He et al., 2019; Slater-Hammel, 1960). The SST has been used in the vast majority of response inhibition research in cognitive psychology (Senkowski et al., 2024; Verbruggen & Logan, 2008a, 2008b). Lately, this research paradigm has also drawn considerable attraction in the field of sport psychology (e.g., Albaladejo-Garcia et al., 2023; Fleddermann et al., 2023; Hagyard et al., 2021), where the SST has been used to examine expert-novice differences in response inhibition across different sports. Recent reviews show that expert athletes display superior motor inhibition performance compared to novices (Simonet et al., 2023; Albaladejo-Garcia et al., 2023). These expertise effects have been reported for different team-sports, like volleyball (Meng et al., 2019), handball (Heppe & Zentgraf, 2019), rugby, soccer, and basketball (Hagyard et al., 2021). In addition, the expertise level appears to influence the inhibition performance of elite athletes. Elite athletes with higher expertise outperform elite athletes with lower expertise (Fledermann et al., 2023). With regards to the type of sport skill, it seems that response-inhibition performance benefits from practicing open-skill sports as compared to practicing closed-skill sports (Wang et al., 2013; Zhu et al., 2020).

Importantly, in the SST, the timing of the action is uncertain, as participants (only) respond after an (unpredictable) go-signal and thus, response initiation is based on *reaction*. In contrast, in ARI tasks, the timing of the action is certain, as participants respond to a (predictable) go-signal and thus, response initiation is based on *anticipation*. The ARI task was originally introduced by Slater-Hammel (1960), where participants viewed a clock, with a clock hand starting from the twelve o’clock position and revolving in one second to complete a full circle. The participants were asked to stop the revolving clock hand exactly at the eight o’clock position by releasing a response button (go-trials). In these go-trials, they stopped the clock hand on average with a constant error of 26 ms after the eight o’clock position. In some trials, however, the clock hand stopped before it reached the eight o’clock position, and in these cases, participants had to inhibit the response (stop-trials). For these stop-trials, it was found (1) that participants inhibition performance decreased the closer the clock hand came to the eight o’clock position and (2) that after a certain point in time (i.e., after the PNR), participants were not able to inhibit their prepared response anymore and initiated the action, although the clock hand stopped moving (and did not reach the eight o’clock position). In the study of Slater-Hammel (1960), the PNR was 166 ms before the eight o’clock position. This pattern of results reported by Slater-Hammel (1960) was later replicated by Coxon et al. (2006) with the PNR at 183 ms and by Carlsen et al. (2008) with the PNR at 140–150 ms before the target. With regard to the pump fake in basketball mentioned earlier, the ARI task may be a more suitable paradigm to investigate the PNR for the defender during the throwing action of the attacker than the SST, because the defensive action of trying to block the jump shot is rather based on anticipation than on reaction.

The use of domain-specific paradigms to examine response inhibition, which reflect the requirements of complex decision making in sports, is still rare. Most of the previous studies on inhibitory control in sport psychology mentioned above used standard forms of the “classic” SST without any adaptation to the sports context (e.g., Fleddermann et al., 2023; Heppe & Zentgraf, 2019; Meng et al., 2019). While these studies are certainly valuable contributions to the sport psychological research field, the external validity of the results is only limited when participants respond to oversimplistic stimuli. For the ARI task, however, there has been a rare exception with one sport-specific adaptation looking at response inhibition in baseball batting (Gray, 2009). Therefore, the present study has been designed to develop a further sport-specific paradigm with the aim to investigate reponse inhibition for the jump shot in basketball. Because the defensive action of trying to block the jump shot is rather based on anticipation than on reaction, we designed a basketball-specific ARI task (instead of using the “classic” SST) and isolated the PNR at which participants were no longer able to inhibit their inappropriate responses in two experiments. This will inform us about the limits of action control in dynamic sport-specific environments, requiring complex decision making in real-life situations (Abernethy et al., 1994; Kalén et al., 2021; Nakamoto & Mori, 2008; Williams et al., 2008).

## Experiment 1

Even though successful defensive actions in basketball both depend on timely action initiation (i.e., in case of a jump shot) and on action inhibition (i.e., in case of a pump fake), the ability to suppress actions volitionally has not yet been studied experimentally in a basketball-specific context. Accordingly, Experiment 1 was conducted to validate a set of basketball-specific stimulus material of the jump shot to investigate participants’ response inhibition skills. To this end, the paradigm of Slater-Hammel (1960) was modified to isolate the PNR, to determine the limits of action control in an ARI task for these basketball-specific stimuli. Participants viewed a video of a basketball player performing a jump shot and their task was to time the response to “block” the shot exactly when the ball leaves the player’s fingertips (go-trials) by releasing the space bar. In 25% of the trials, however, the video stopped prematurely before ball release and participants were asked to withhold the response (stop-trials).

In line with previous studies using the ARI task (e.g., Carlsen et al., 2008; Coxon et al., 2006; Slater-Hammel, 1960), it is expected that participants’ inhibition performance decreases the closer the video stops before the ball leaves the player’s fingertips (Hypothesis 1). This should be the case as the signal to inhibit the action (i.e., the stop of the video) appears later in relation to the point of ball release and, as a consequence, the action may not be suppressed successfully because there is not enough time to cancel the prepared motor response.

In previous anticipation-response-inhibition tasks (e.g., Leunissen et al., 2017; Slater-Hammel, 1960), simple stimuli (e.g., a revolving clock handle or bars that are being filled) moving with a constant velocity were used. In the present study, however, participants have to inhibit their responses based on the anticipation of a movement with accelerated velocity displayed in a sport-specific environment. Specifically, the ball’s velocity during the throw is accelerated when being moved by the attacker on a trajectory from hip height to the point of ball release at the end of the jump shot. At first glance, response inhibition in this real-life situation seems to be more difficult. However, previous research has found that more complex sport-specific stimuli seem to have similar processing demands as simple, static stimuli that have been used in “classic” SSTs (Ko et al., 2016; Scharfen et al., 2022). Also, responses in a coincidence anticipation task (with the aim of target interception) can be as precise for targets travelling with constant velocity as for targets with accelerated velocity (Fialho & Tresilian, 2017). Therefore, it is hypothesized that the location of the PNR found in previous studies using more simple stimulus displays (e.g., Slater-Hammel, 1960; Coxon et al., 2006; Carlsen et al., 2008) can be replicated when using basketball-specific stimuli (Hypothesis 2).

Due to the different conditions of the experiment (go-trial vs. stop-trial), participants must balance between responding as precisely as possible in a go-trial and trying to inhibit their response in a stop-trial. Therefore, participants must adjust their response threshold based on the expectation of the next trial being a stop-trial or a go-trial. When participants shift their priority from the go-trial to the stop-trial, responses are prolonged (resulting in a larger constant error [CE]) and the PNR is reduced, which is an indication for an attentional change. These adjustments can be proactively, based on the overall expectation of stop-trials within an experiment but also on a trial-to-trial basis, by displaying a pre-cue before a stop-trial (Verbruggen & Logan, 2009) or reactively in a go-trial after a previous stop-trial was presented (Bisset & Logan, 2011). Whether those reactive adjustments occur after a stop-trial or only after an unsuccessful inhibition is under debate. While Rieger and Gauggel (2010) found strategic adjustments after a stop-trial, Verbruggen and Logan (2008b) only found those adjustments after an unsuccessful inhibited stop-trial. To this end, it is expected that participants delay their response in the present go-trial after a stop-trial had occurred in the previous trial (Hypothesis 3), which reflects a strategic adjustment based on the previous trial history. Whether this kind of adjustment only occurs after an unsuccessful stop-trial or in general will be investigated in an explorative way.

### Methods

#### Participants

The study was assessed as ethically noncritical by the Ethics Committee of Paderborn University. All data were saved, analyzed, and published anonymously. For calculating the sample size, G.Power 3.1.9.7 was used (Faul et al., 2007). For a main effect of the within-subject factor *go-trial n-1* (go-trial, unsuccessful stop-trial, successful stop-trial) of f = 0.25, and an α-value of 0.05, at least 24 participants were necessary to reach a power of 0.90. Rieger and Gauggel (2010) and Verbruggen et al. (2008) both reported large effect sizes (d = 1.2; n2p = 0.37) for the within factor trial n-1. However, because we modified the paradigm with regard to the stimulus material, we chose a more conservative effect size for sample size calculation. In total, twenty-seven sport science students participated. Participation was voluntarily and participants signed an informed consent form prior to the experiment. They received course credits, but no financial reward. All participants had normal or corrected visual acuity and had no psychopathological and neurological disorders. Twenty-two participants were right-handed and five were left-handed. None of them played basketball on a club level or watched basketball regularly, however, some had gained practical experience at school or during a basketball course within the sport science study program. Test data from two participants were excluded because they disregarded the instructions. Instead of trying to be as accurate as possible in the go-trials, they delayed their responses while waiting for a stop-trial, resulting in an extremely high constant error and go-mission rate. Therefore, a correct determination of the PNR was not possible, since nearly all stop-trials could be inhibited and there was no opportunity to fit the data into a psychometric function. After the exclusion of these two participants, the data of the remaining twenty-five participants (male = 15, female = 10, *M*_*age*_ = 22.0) were analysed.

#### Apparatus and stimulus

For stimulus material (see Fig. 1), a video of a male basketball player performing a jump shot was recorded with a Canon Legria HF G40 with 50 fps from a defensive player’s perspective (face-to-face, front view perspective). The player recruited for the video played basketball for about 20 years but was not an active player at the time of recording anymore. The highest league he used to play in was the first regional league in North Rhine-Westphalia, with a basketball specific training time of six hours per week. The recording took place in a gymnasium. The basketball player was wearing a blue t-shirt and black shorts. He was positioned in front of the basket and the camera was placed across at the baseline underneath the basket. At the beginning of the recording, the basketball player looked into the camera and held the ball centered in front of his body. The basketball player’s feet had firm contact to the ground, the knees and hips were slightly bend, and the upper body was upright. Half of the right foot was placed in front of the left one and the right shoulder slightly pointed to the basket. The right (throwing) hand was placed on top of the ball and the left (supporting) hand secured the ball from the side (starting position). After the recording started, the basketball player bounced the ball with his right hand for three times (bouncing completed 1850 ms after the video started). After bouncing, he bent his knees and hips, while concurrently moving the throwing hand behind the ball and raising his arm until the back of the right hand was placed above his forehead (ball above the player’s forehead 2400 ms after the video started). Afterwards, he extended his knees, hip, and ancles (triple-extension) to perform a vertical jump. At the same time of the triple-extension, the player lifted the shoulder of the throwing hand and extended his right elbow to transmit the power of the triple-extension to the throwing hand and ball. As his right arm was fully extended, he hinged his wrist downwards to rotate the ball backwards. The ball left the fingertips of the right hand at the highest point of the jump with the throwing arm pointing towards the basket (so-called follow through) (ball leaving the fingertips 2716 ms after the video started).

For better experimental control of the whole action displayed in the stimulus, the video was edited manually. First, the video was cut to a total length of 180 video frames (i.e., a length of 3000 ms). Second, all visual content not relevant for action perception, such as signs and posters placed on the wall in the background of the video, was erased to avoid any unnecessary distraction of attention. Third, to mark the point, where the ball leaves the fingertips, a black circle was inserted (and shown throughout the whole video), with the ball fitting exactly into the circle when it “passed through” (i.e., 2716–2733 ms after the video started; see Fig. 1a, second last frame on the far right). The video stimulus had a size of 929 × 1035 pixel and was presented on a computer screen, with a size of 24 inches, a resolution of 1080p, and with a refresh rate of 60 Hz. The presentation of the image sequence was controlled by ©Presentation 22.1. The single video images were updated every refresh rate (60 Hz = 16.7 ms). Due to the slightly different frame rate of the recorded video and the refresh rate, the video speed was accelerated by about 3.33 ms (10 Hz) per frame. We chose a higher refresh rate for a more realistic execution time and a higher precision of determining the point-of-no-return (PNR).

#### Procedure

After participants signed the informed consent form, they completed a short questionnaire to collect demographic data and sport specific information, such as age, sex, corrected visual acuity, psychopathological and neurological disorders (cf. Verbruggen & Logan, 2008a), basketball expertise, the sport being practiced, and the training frequency in hours per week. Afterwards, participants received written and verbal instructions about the experimental task and how they had to respond in the stop- and go-trials. Before the experiment started, participants were verbally instructed to sit upright, place their feet on the ground with solid contact, and to place their arms on the table with the elbows bend at about 90 degrees. The keyboard was placed centred to the body in an easily reachable distance. To start a trial, participants were instructed to press (and hold) the spacebar. After the spacebar was pressed, a fixation cross was presented at ball’s height for 500 ms, whereupon the video sequence started. Participants were instructed to release the spacebar exactly at the point, where the ball leaves the fingertips (go-trials) or to withhold their response when the video stopped prematurely (stop-trials). Go-trials occurred in 75% and stop-trials in 25% of all trials, respectively. For the stop-trials, the delay between the stop and the point of ball release was adjusted by a staircase-tracking algorithm based on participants’ performance in the previous trial with a fixed step size of 16.7 ms, which corresponds to one video frame. Accordingly, if a participant successfully inhibited his/her response in the previous stop-trial, the time interval between the point of ball release and the premature stop of the video was decreased for the next stop-trial by one video frame (i.e., the time interval became smaller and thus, the video stop-delay moved closer to the point of ball release), and, vice versa, was increased by one video frame, if the participant was not successful to inhibit the response (i.e., the time interval became larger and thus, the video stop-delay moved away from the point of ball release). Using this staircase-tracking algorithm has the advantage of providing a more accurate and reliable estimate of the PNR, since (overall) there are more attempts located near the PNR (Levitt, 1971). In this regard, the initial stop was set to 200 ms before the point of ball release for the first stop-trial of the experiment, based on the results of a pilot testing. The experiment consisted of three test blocks of 200 trials each, resulting in 600 trials in total. The trials varied with regard to the type of trial (go-trial vs. stop-trial) and were presented in a randomized order, generated by the Presentation software. We chose a high percentage of go-trials (75%) to avoid the strategy of participants to wait for stop-trials and this way, to increase the readiness to respond to go-trials for an increased response precision (Verbruggen & Logan, 2009; Verbruggen et al., 2019). Thus, go-trials were repeated 150 times and stop-trials 50 times within a test block. To become familiar with the task, participants completed a practice block of 20 trials (15 go-trials, 5 stop-trials) prior to the start of the experiment. Only in the practice block, participants received feedback 1000 ms after each trial, for 2000 ms. After a go-trial, participants received feedback about their precision (i.e., constant error). If participants stopped the video before the target image, they received feedback “zu früh” ["too early” in German]); if participants stopped the video after the target image, they received feedback “zu spät” [“too late” in German]). After a stop-trial, participants received feedback about the correctness of their response. If participants were able to inhibit their response in a stop-trial, they received the feedback “korrekte Antwort” [“correct answer” in German]; if they were not able to inhibit their response, they received the feedback “falsche Antwort” [“incorrect answer” in German]. If the participants responded before the video started, they received feedback “fehlende Antwort” [“missing response” in German] and if they did not respond in a go-trial, they received feedback “Antwort zu früh” [“early response” in German] 1000 ms after the trial for 2000 ms. No feedback on precision and correctness was given after the practice block.

After each block, including the practice block, participants received feedback about their overall performance in the current block. In line with the study by Verbruggen and colleagues (Verbruggen et al., 2019), the performance feedback included (1) participant’s mean anticipation response accuracy, provided as constant error (CE; i.e., reflecting the average directional error relative to the point of ball release), (2) the mean probability of responding to a stop-trial (false alarm), (3) and the total number of go-trials in which no answer was given (go-omissions). The constant error was calculated by subtracting the time the ball left the fingertips from the response time [CE = (X – 2733 ms) ms^1^]. After each test block, participants were allowed to take a break. The entire experiment lasted between 70 and 80 min.

### Statistical analysis

To determine the ability of response inhibition, the PNR was calculated by averaging the peaks and valleys of all runs for every participant (Wetherill, 1963). A run was defined as a series of increasing or decreasing time interval in one direction. Thus, a peak is the delay that is the last value of an ascending series of time intervals before the participant can no longer suppress his/her response and the stop-delay therefore decreases again. A valley is the delay that is the last value of a descending series of time intervals before the participant can suppress his/her response again and the stop-delay increases again. Following from this, the calculated PNR was used as a dependent variable and was analyzed regarding the within-subject factor *block* (Block 1–3) by a repeated-measures ANOVA in order to detect potential practice effects that could have an influence on the ability of inhibition. To measure response precision, the total number of hits and the constant error were calculated. To identify possible practice effects regarding response precision as well, two additional repeated-measures ANOVAs were conducted. The calculated total number of hits and the constant error were used as dependent variables respectively, and both were analyzed regarding the within-subject factor *block* (Block 1–3). In addition, a repeated-measures ANOVA was conducted to investigate whether a stop-trial influences the response accuracy in a subsequent go-trial. Accordingly, the constant error was analyzed regarding the within-subject factor *go-trial n-1* (go-trial, successful stop-trial, unsuccessful stop-trial). Prior to the analysis, the data were checked, and incorrect answers (i.e., trials where participants lifted their finger off the response key before the video had started or did not respond at all) were excluded. For all analyses, the α-value was set to 0.05 and the effect size was calculated using the partial eta-square. In case of significant main effects, post-hoc t-tests were conducted. If any violations of sphericity occurred, the Greenhouse–Geisser adjustment was used for correction. All statistical analyses were performed using ©IBM SPSS Statistics 29.

### Results

#### Inhibition performance

**Fig. 2:**
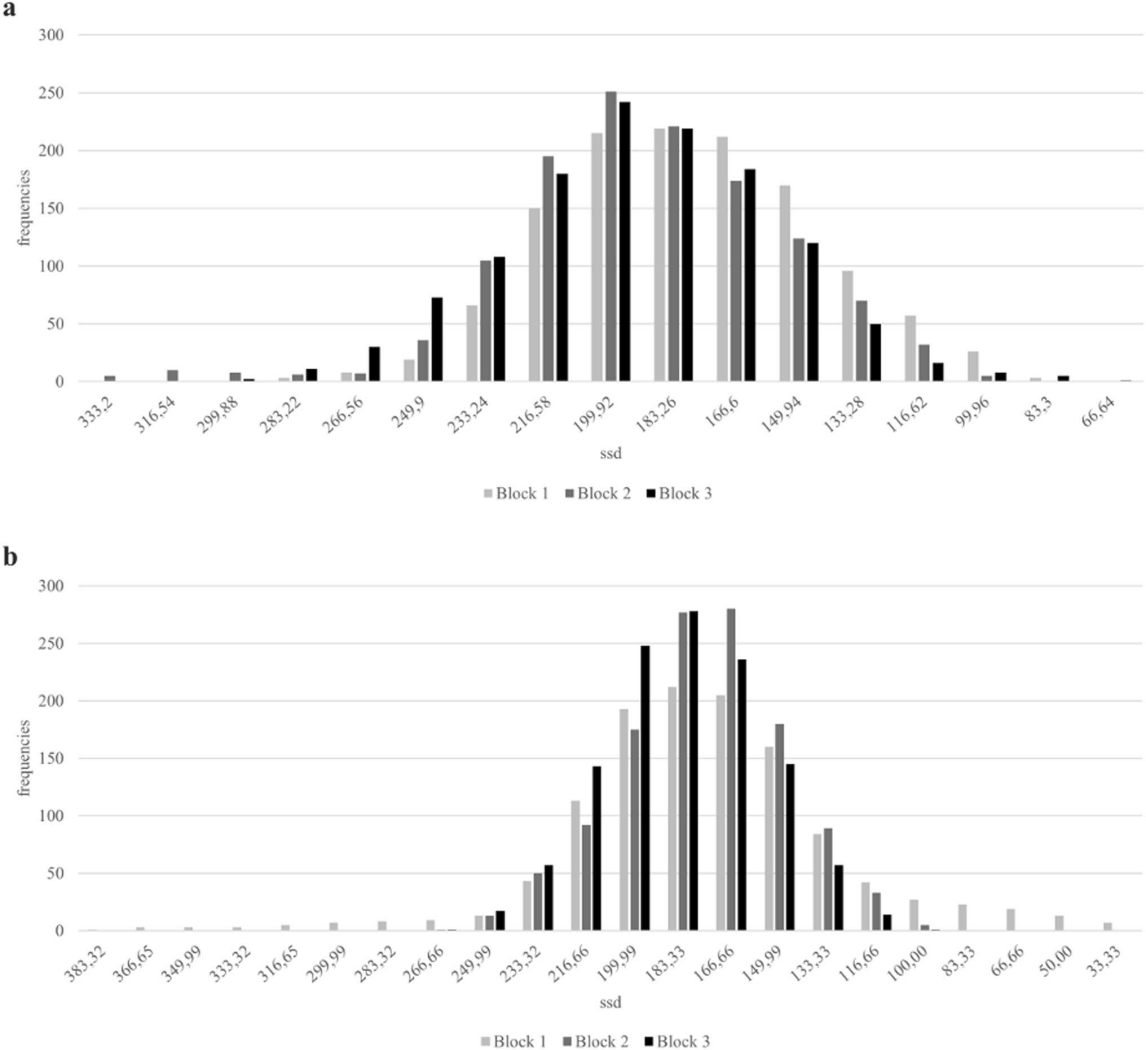
Frequency distribution of stop-delays across blocks. Note: Results for Experiment 1 (**a**) and Experiment 2 (**b**)

**Fig. 3:**
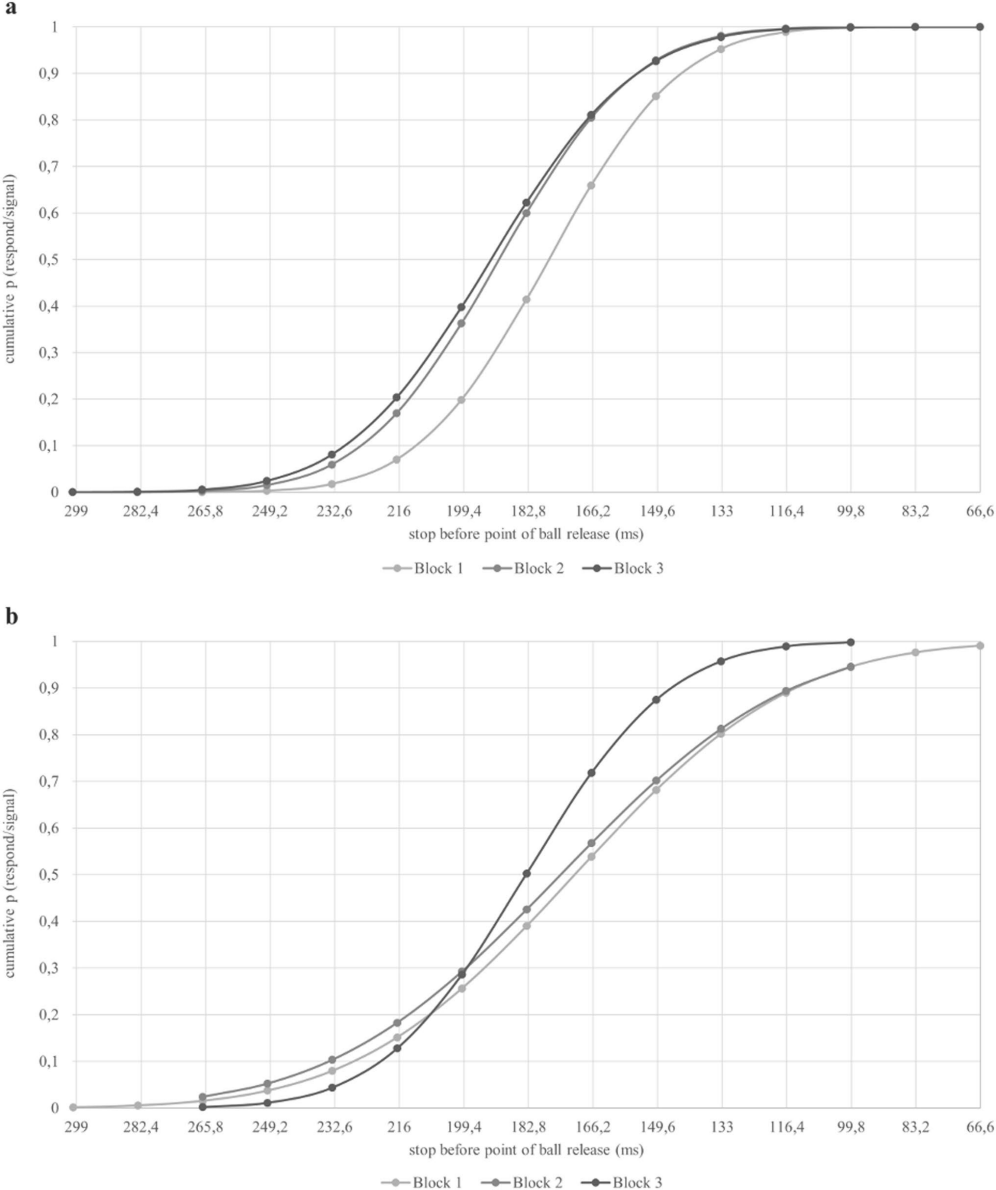
Cumulative function of the probability to respond in a stop-trial in each block. Note: Results for Experiment 1 (**a**) and Experiment 2 (**b**)

By using the staircase-tracking algorithm, the average response probability should be close to 50% (cf. Verbruggen et al., 2019). In Experiment 1, participants were able to inhibit their response on average in 49.36% (*SEM* = 0.38) of all stop-trials. There was a main effect for the factor *block* [*F*(2.48) = 7.547, *p* < 0.001; *ɳp2* = 0.239].The mean probability in Block 1 (*M* = 48.68%, *SEM* = 0.46) was not significantly lower than in Block 2 (*M* = 49.32%, *SEM* = 0.43, *p* = 0.069), but significantly lower than in Block 3 (*M* = 50.08%, *SEM* = 0.391, *t*(24) =  – 3.742, *p* < 0.001, *d* =  – 0.748). Performance in Block 2 was not different from Block 3 (*p* = 0.052). Figure 2a shows the frequency distribution of stop delays throughout Experiment 1, separately for each block. The stopping times throughout the experiment ranged from 333 to 67 ms before the point of ball release. The peak values also correspond to a large extent to the inhibition probability as a result of the staircase-tracking algorithm. Since the time interval between the point of ball release and the premature stop of the video was adjusted based on participants’ performance, most of the stop-delays were around the mean PNR. A more detailed explanation of the staircase-tracking algorithm is provided in the section “procedure”. The PNR was calculated according to Wetherill (1963) and was located at 187 ms before the point of ball release. The PNR and associated inhibitory performance decreased across blocks [*F*(1.625, 39.001) = 11.659, *p* < 0.01; *ɳp2* = 0.327] (see Fig. 3a). To evaluate the main effect for the factor *block*, paired t-test were conducted. Unexpectedly, the PNR in Block 1 was significant later (i.e., closer to the point of ball release, *M* = 177.46, *SEM* = 5.33) compared to Block 2 (*M* = 190.48, *SEM* = 5.53, [*t*(24) = -3.81, *p* < 0.001, d = 0.762]) and Block 3 (*M* = 192.19, *SEM* = 5.90, [*t*(24) = 3.745, *p* < 0.001, *d* = 0.749]), as also shown in Table 1. No significant difference was found between Block 2 and Block 3 (*p* = 0.502).

**Table 1:** Means and standard error of the mean (brackets) for response and inhibition rates

	Block 1	Block 2	Block 3	Mean
Experiment 1				
PNR (ms)	177.5 (5.3)	190.5 (5.5)	192.2 (5.9)	186.7 (5.3)
p(respond/stop) (%)	48.7 (0.5)	49.3 (0.4)	50.1 (0.4)	49.4 (0.4)
constant error (ms)	16.8 (4.9)	11 (4.3)	1.3 (3.35)	9.6 (0.7)
hits (total)	22.1 (2.0)	27.0 (2.4)	30.5 (2.1)	26.5 (2.1)
go-trial/go-trial (ms)	10.3 (4.8)	4.9 (4.4)	-2.1 (3.1)	4.3 (3.8)
successful stop-trial/go-trial (ms)	28.5 (6.1)	20.3 (4.8)	13.3 (4.2)	20.6 (4.4)
unsuccessful stop-trial/go-trial (ms)	26.0 (5.3)	16.4 (5)	– 2.6 (9.2)	13.8 (5.1)
Experiment 2				
PNR (ms)	171.9 (6.6)	176.2 (4.2)	183.3 (3.8)	177.4 (0.8)
p(respond/stop) (%)	47.5 (0.6)	48.3 (0.4)	49.3 (0.3)	48.5 (0.3)
constant error (ms)	26.7 (6.1)	12.7 (3)	8.0 (2.5)	15.7 (0.5)
hits (total)	17.7 (1.5)	25.1 (1.6)	31.4 (1.9)	24.7 (1.2)
go-trial/go-trial (ms)	21.1 (5.5)	7.7 (3)	4.7 (2.3)	11.0 (3.1)
successful stop-trial/go-trial (ms)	45.6 (8.4)	28.1 (3.8)	19.9 (3.4)	31.0 (4.5)
unsuccessful stop-trial/go-trial (ms)	45.4 (7.7)	28.3 (4.7)	16.9 (3.9)	29.8 (4.6)

#### Precision

In the go-trials, participants stopped the video at the exact point where the ball left the fingertips (hits) in 26.5 (*SEM* = 2.08) go-trials out of the 450 total go-trials. Repeated-measures ANOVA revealed a main effect for the factor *block*, as the number of hits [*F*(2, 48) = 28.88, *p* < 0.001; *ɳp2* = 0.546] increased across blocks (see Table 1). The follow-up paired t-tests showed that the number of hits in Block 1 was significantly lower than in Block 2 [*t*(24) =  – 4.045, *p* < 0.001, *d* = 0.809] and Block 3 [*t*(24) =  – 8.481, *p* < 0.001, *d* = 1.696)]. The number of hits in Block 2 was significantly lower than in Block 3 [*t*(24) =  – 3.131, *p* < 0.005, *d* = 0.626]. Furthermore, the participants delayed their responses in the go-trials by an average of 9.6 ms (*SEM* = 0.7). Repeated-measures ANOVA revealed a main effect for the factor block, as the participants precision performance (i.e. smaller CE) increased across blocks [*F*(2, 48) = 15.023, *p* < 0.001; *ɳp2* = 0.385]. Paired t-tests showed, that the CE in Block 1 was significantly higher than in Block 2 [*t*(24) = 2.209, *p* = 0.018, *d* = 0.442] and Block 3 [*t*(24) = 4.651, *p* < 0.001, *d* = 0.930)] and the CE in Block 2 was significantly higher than in Block 3 (*t*(24) = 3.819, *p* < 0.001, *d* = 0.764].

#### Post-stop-trial adjustments

**Fig. 4:**
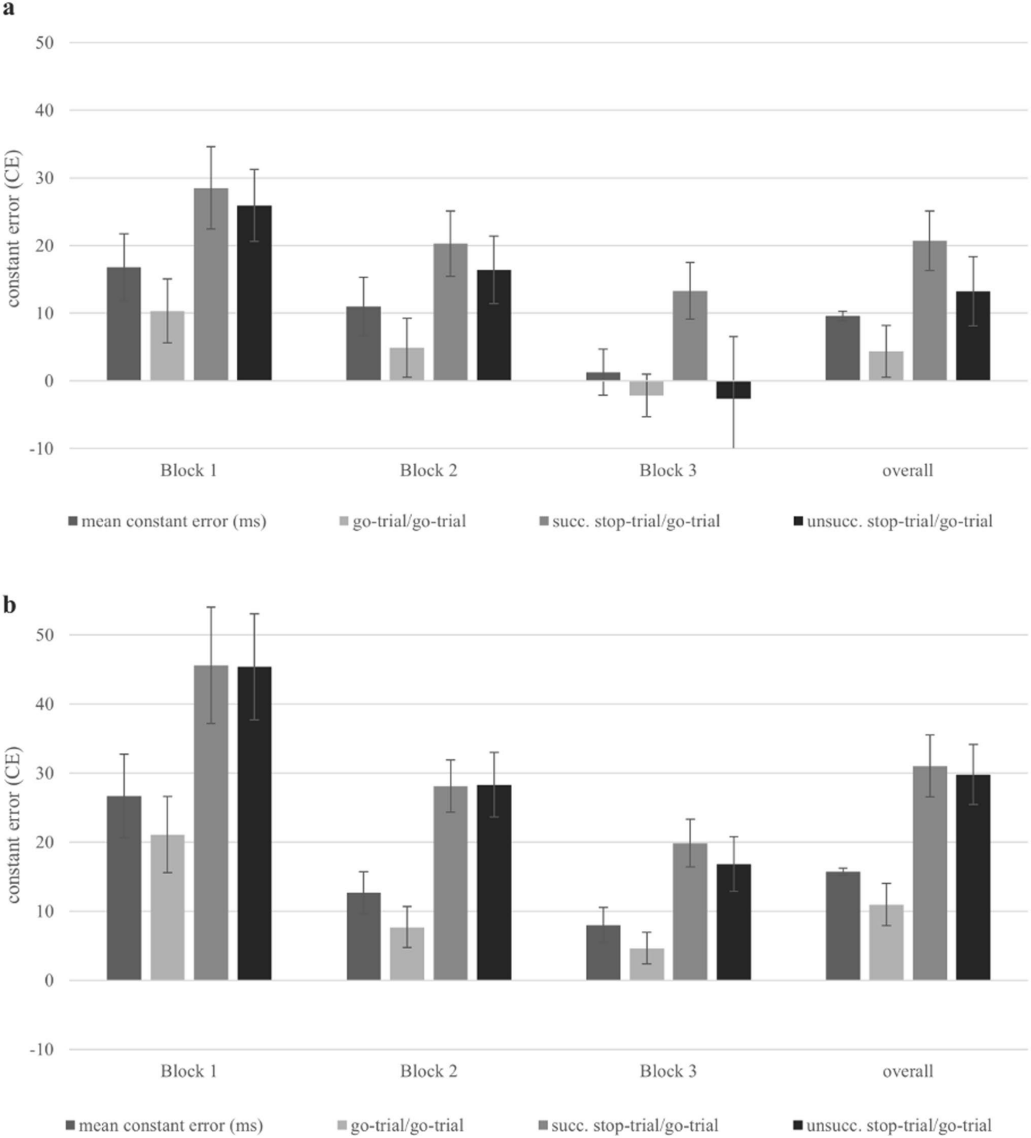
Constant error (CE) and post-error adjustments in each block and overall. Note: Results for Experiment 1 (**a**) and Experiment 2 (**b**). CE in a go-trial and in a go-trial in trial n after a go-trial (go-trial/go-trial), after an unsuccessful stop-trial (unsuccessful stop-trial/go-trial), and after a successful stop-trial (successful stop-trial/go-trial). Error bars show the standard error of the mean

To determine the impact of a stop-trial on participant’s precision in a subsequent go-trial, a repeated-measures ANOVA was used. The repeated measures ANOVA showed a main effect for the factor *go*-*trial n-1* [*F*(2, 48) = 17.865, *p* < 0.001; *ɳp2* = 0.427]. Paired t-tests were conducted to evaluate the differences for the factor *go-trial n-1*. The precision, measured by the CE, in a go-trial after a successful stop-trial [*t*(24) =  – 7.457, *p* < 0.001, *d* =  – 1.491] as well after an unsuccessful stop-trial [*t*(24) =  – 3.205, *p* = 0.002, *d* =  – 0.641] was higher than after a go-trial in a consecutive go-trial (see Fig. 4 and Table 1). Furthermore, the success of inhibition in a stop-trial affected the size of the CE [*t*(24) = 2.268, *p* = 0.016, *d* = 0.439].

### Discussion

Experiment 1 was conducted to validate a set of basketball-specific stimulus material to investigate response inhibition for the basketball jump shot. Therefore, the experimental paradigm of Slater-Hammel (1960) was modified to experimentally isolate the PNR, to determine the limits of action control in an anticipation-response-inhibition task with basketball-specific stimuli. To this end, participants watched a video of a basketball player executing a jump shot and were asked to release the response button as accurately as possible at the point where the ball leaves the basketball player's fingertips (go-trials). In a subset of the trials (stop-trials), however the video stopped before ball release and participants were asked to withhold their finger-lifting response. By using the staircase-tracking algorithm, which decreased or increased the time interval between the stop and the point of ball release, in the following stop-trial, depending on the success of inhibition, a wide range of stop delays (i.e., time interval between video stop and point of ball release) was found, showing that inhibition ability varies widely across blocks and across the experiment.

As assumed in Hypothesis 1, the further away the stop was from the point where the ball left the fingertips, the more likely participants were able to inhibit their responses, and, vice versa, the closer the stop was to the point of ball release, the less likely they were able to inhibit their answers (e.g., Carlsen et al., 2008; Coxon et al., 2006; Slater-Hammel, 1960). The decrease in inhibition probability proves that the time to stop the action decreases and after a certain point is no longer sufficient to successfully stop the execution of the action. The PNR was located 187 ms before the point of ball release, which corresponds to previous results and confirms Hypothesis 2. Interestingly, the PNR was found later (i.e., closer to the point of ball release) in Block 1 compared to Block 2 and Block 3 (Fig. 3), which appears to be the opposite trend from what would be expected for a practice effect. However, when considering the results for the CE and the total number of hits across the three blocks (Table 1), it becomes evident that participants increased precision performance in terms of target anticipation (i.e., the exact point when the ball leaves the player’s fingertips). This most likely reflects a strategic adaptation of the response behavior over the course of the whole experiment in favor of precision over inhibition performance. The latter is also a confirmation for choosing a high percentage of go-trials (i.e., 75%) to avoid the strategy of participants to wait for stop-trials and this way, to increase the readiness to respond to go-trials for an increased response precision, which is in line with previous studies (Verbruggen & Logan, 2009; Verbruggen et al., 2019).

Another strategic adaptation of the response behavior was of interest. In accordance with previous studies (Rieger & Gauggel, 2010; Verbruggen & Logan, 2008b; Verbruggen et al., 2008), it was assumed that participants delay their responses in a go-trial after a stop-trial occurred in the previous trial, which reflects a strategic adjustment based on the experience to inhibit the action in the previous trial (Hypothesis 3). The results confirmed that participants strategically delayed their responses after a stop-trial by adjusting their response behavior, depending on whether the response was successfully inhibited or not. After successful inhibition, the response delay was higher than after an unsuccessful inhibition.

## Experiment 2

In the first step, the validation of the set of sport-specific stimulus material of the basketball jump shot was successful in Experiment 1. However, it is not realistic that the jump shot in basketball is simply interrupted during the real game. Instead, players often use a pump fake as a deceptive action to trick the opponent into a wrong reaction before they attempt the jump shot. In a second step, the stimulus material was therefore further modified to provide a more realistic response scenario in Experiment 2. To this end, the video was stopped at a certain point before the ball left the fingertips of the attacker, paused for 50 ms, and then played backward until the ball was at hip height again. This way, we were able to present a video simulation of a pump fake and thus, examine the limits of actions control for response inhibition of a deceptive action in an experimentally controlled manner. If the pattern of results in Experiment 2 were similar to the ones observed in Experiment 1, then this would suggest that the limits of action control for response inhibition in the basketball scenario generalize from an interrupted action (i.e., the stopped jump shot) to a deceptive action (i.e., the pump fake). In this case, the sport-specific stimulus material would be suitable to further investigate the ability to inhibit complex responses in basketball, such as a blocking action to a pump fake.

### Methods

#### Participants

The Ethics Committee of the Paderborn University assessed the study as ethically noncritical. All data were saved, analyzed, and published anonymously. The sample size was calculated with MorePower 6.0.4. In Experiment 1, main effects for the within-subjects factor *block* (Block 1–3) with respect to the dependent factors PNR, constant error, and hits were found. For a conservative sample size estimation, the smallest effect size for the main effect for the within-subjects factor *block* was used, which was found with respect to the dependent factor PNR (n2p = 0.24). Consequently, to achieve a power of 0.90, a minimum of 22 participants are required for a main effect for the within-subjects factor *block* of f = 0.562 and an *α*-value of 0.05. The participants participated voluntarily in the study, without any financial reward, but received course credits. Prior the experiment an informed consent had to be signed. No participant had abnormal or uncorrected visual acuity nor any psychopathological and neurological disorders. None of the participants were experienced basketball players. Some had practical experience at school or in practical seminars attended as a part of their studies. A total of 24 sport science students (male = 14, female = 10) aged between 18 and 29 years (*M*_*age*_ = 21.75) participated. 21 participants were right-handed, the other ones were left-handed.

#### Apparatus und stimulus

The apparatus and the stimulus material used in Experiment 2 were the same as in Experiment 1.

#### Procedure

Participants signed the informed consent form and completed a short questionnaire to collect demographic data and sport specific information before the experiment started. The written and verbal instructions of the body placement and about the experimental task and how they had to respond in the stop- and go-trials were given before the testing started (for more details on the instructions and data collection, see Experiment 1). As in Experiment 1, participants were instructed to lift their finger from the space bar when the ball leaves the player's hand, which was marked with a black circle (go-trials). In the stop-trials, however, the video was not merely stopped at a certain point before the point of ball release, but briefly paused for 50 ms and then played backward until the ball was at hip height again (Fig. 1c), to simulate the pump fake in basketball.

The whole experiment consisted of three test blocks of 200 trials each, resulting in a total of 600 trials. After each test block, participants were allowed to take a break. The distribution on 75% go-trials and 25% stop-trials remained the same and were presented in a randomized order, generated by the Presentation software. The delay between the reversal point and the point at which the ball was released was adjusted by a staircase-tracking algorithm based on participants' performance in the previous trial with a fixed step size of 16.7 ms. A practice block with 20 trials (15 got-trials, 5 stop-trials) was completed before the testing started, to become familiar with the task. Only in the practice block, participants received feedback 1000 ms after each trial, for 2000 ms. After each block, including the practice block, participants received feedback about their overall performance in the current block. It should be noted here that the calculation of the constant error in Experiment 2 was adjusted by using the onset of the presentation time of the target position [CE = (X – 2716 ms) ms].^2^

### Statistical analysis

The same statistical analyses were used as in Experiment 1. The calculated PNR and the total number of hits as well as the constant error were used as dependent variables and were each analyzed with regard to the within-subject factor *block* (Block 1–3) by a repeated-measures ANOVA to identify potential practice effects. To examine post-stop-trial adjustments, the dependent variable constant error was analyzed with respect to the within-subject factor *go-trial n-1* (go-trial, successful stop trial, unsuccessful stop trial), using a repeated-measures ANOVA. Prior to analysis, data were checked, and incorrect responses (i.e., trials where participants lifted their finger off the response key before the video had started or did not respond at all) were excluded. For all analyses, the α-value was set to 0.05 and the effect size was calculated using partial eta-square. For significant main effects, post-hoc t-tests were performed, and the Greenhouse–Geisser adjustment was used if violations of sphericity occurred. For all statistical analyses, ©IBM SPSS Statistics 29 was used.

### Results

#### Inhibition performance

Participants were able to inhibit their response on average in 48.53% (*SEM* = 0.271) of all stop-trials. There was a main effect for the factor *block* [*F*(1.519, 31.891) = 4.014, *p* = 0.038; *ɳp2* = 0.152], indicating that the blocks were significantly different from each other. To further evaluate the main effect for the factor *block*, paired t-tests were conducted. The mean probability in Block 1 (*M* = 47.77%, *SEM* = 0.6, *t*(21) =  – 2.375, *p* < 0.014, *d* =  – 0.506) and in Block 2 (*M* = 48.45%, *SEM* = 0.36, *t*(21) =  – 2.373, *p* < 0.014, *d* =  – 0.506) were significantly lower than in Block 3 (*M* = 49.36%, *SEM* = 0.36,). The mean probabilities in Block 1 and Block 2 did not differ significantly (*p* = 0.113). Figure 2b shows the frequency distribution of stop delays throughout Experiment 2. The stopping times throughout the experiment ranged from 383 to 33 ms before the point of ball release. The peak values also correspond to a large extent to the inhibition probability as a result of the staircase-tracking algorithm. Since the time interval between the point of ball release and the premature stop of the video was adjusted based on participant’s performance, most of the stop-delays were around the mean PNR. The PNR was calculated according to Wetherill (1963) and was located at 177 ms before the point of ball release. The PNR and associated inhibitory performance did not decreased across blocks [*F*(1.219, 28.032) = 2.256, *p* = 0.140] (see Fig. 3b).

#### Precision

Participants stopped the video at the exact point where the ball left the fingertips (hits) in 24.7 (*SEM* = 1.16) go-trials out of the 450 total go-trials. Repeated-measures ANOVA revealed a main effect for the factor *block*, as the number of hits [*F*(1.601, 36.822) = 33.437, *p* < 0.001; *ɳp2* = 0.592] increased across blocks (see Table 1). Paired t-tests show that the number of hits in Block 3 was significantly higher than in Block 2 [*t*(23) = 4.642, *p* < 0.001, *d* = 0.948)] and Block 1 [*t*(23) = 6.711, *p* < 0.001, *d* = 1.370]. Also, the number of hits in Block 2 was significantly higher than in Block 1 [*t*(23) = 4.748, *p* < 0.001, *d* = 0.969]. In addition, the constant error show that participants delayed their responses in the go-trials by an average of 15.7 ms (*SEM* = 0.49). Repeated-measures ANOVA revealed a main effect for the factor *block*, as the participants precision performance (i.e. smaller CE) increased across blocks [*F*(1.146, 26.358) = 10.688, *p* = 0.002; *ɳp2* = 0.317]. Paired t-test showed, that the CE in Block 3 was significantly lower than in Block 2 [*t*(23) = 2.382, *p* = 0.013, *d* = 0.486] and Block 1 [*t*(23) = 3.398, *p* < 0.001, *d* = 0.694)]. In Block 2, the CE was significantly lower than in Block 1 [*t*(23) = 3.217, *p* = 0.002, *d* = 0.657] and Block 3.

#### Post-stop-trial adjustments

To determine the impact of a stop-trial on participant’s precision in a subsequent go-trial, a repeated-measures ANOVA was used. The repeated measures ANOVA showed a main effect for the factor *go-trial n-1* [*F*(2, 46) = 65.590, *p* < 0.001; *ɳp2* = 0.740]. A paired t-test was conducted to evaluate the differences for the factor *go-trial n-1*. The precision in a go-trial after a successful stop-trial [*t*(23) = -8.948, *p* < 0.001, *d* =  – 1.826] as well after an unsuccessful stop-trial [*t*(24) =  – 9.995, *p* < 0.001, *d* =  – 2.040] was higher than after two go-trials (see Table 1 and Fig. 4).

### Discussion

Experiment 2 was conducted to further develop the stimulus material and create a more realistic basketball scenario by simulating a pump fake in the stop-trials, instead of only stopping the video of a jump shot at a certain point in time (as in Experiment 1). The results of Experiment 2 are consistent with those observed in Experiment 1. First, the further away the stop was from the point where the ball left the fingertips, the more likely participants were able to inhibit their responses, and, vice versa, the closer the stop was to the point of ball release, the less likely they were able to inhibit their answers. The PNR was found to be 177 ms before the point of ball release, which is comparable to Experiment 1 and to previous studies (Carlsen et al., 2008; Coxon et al., 2006; Slater-Hammel, 1960). An improvement of the PNR across the three blocks of practice was not observed. Second, participants’ precision performance (as measured by the CE) indicated that they responded on an average of 15.7 ms after the point of ball release. However, as in Experiment 1, the CE became smaller and the number of (exact) target hit became larger across the three blocks, reflecting the adaptation of response behavior over the whole experiment in favor of precision performance. Third, a negative influence of a stop-trial on the precision in a subsequent go-trial was found, confirming the strategic adaptation after a stop-trial. However, no influence of the success of inhibition could be detected on the size of the effect. Together, this pattern of results suggests that the limits of action control for response inhibition in the basketball scenario generalize from an interrupted action (i.e., the stopped jump shot in Experiment 1) to a deceptive action (i.e., the pump fake in Experiment 2).

## General discussion

The present two experiments were conducted to investigate the limits of action control on response inhibition for basketball-specific stimulus material by a modification of the experimental paradigm of Slater-Hammel (1960). The results showed that the probability of response inhibition decreases the closer the prepared response gets to the target position (here, the point where the ball is released for a jump shot) and that after a certain point in time, it is not possible to successfully stop the execution of the action. To signify the limits of action control in SST paradigms, the stop-signal reaction time (SSRT) is the standard as a measure of inhibitory performance. In fact, the SSRT can also be measured with the ARI task and may provide a more precise measure as responses are temporally accurate, as recent research has shown (Wadsley et al., 2023; Leunissen et al., 2017). However, whether the processes of response inhibition are the same in the ARI task and the SST has not yet been investigated. Therefore, the term PNR was used in the present basketball-specific ARI tasks to quantify the limits of action control for the basketball-specific stimulus and to distinguish the task from classical SST. The PNR was 187 ms before the ball release in Experiment 1 and 177 ms in Experiment 2. These values for the PNR are only slightly earlier (i.e., reduced inhibition performance) as had been reported in previous studies using simple anticipation-response-inhibition tasks (Carlsen et al., 2008; Coxon et al., 2006; Slater-Hammel, 1960). Thus, different factors of stimulus complexity, including differences of dynamic properties (targets travelling with constant vs. accelerated velocities) may not be decisive for participants’ inhibition performance, as demonstrated before (Ko et al., 2016; Scharfen et al., 2022). Also, short-term practice does not seem to influence the PNR, at least not in a way that participants become better at inhibiting their responses closer to the target, at least not over the course of the two experiments. Instead, a practice effect was observed for participants’ precision performance, as the CE decreased, while the number of (exact) target anticipations increased (Fialho & Tresilian, 2017).

Further, post-stop-trial adjustments indicated that the precision of participants' responses in a go-trial was negatively influenced by a previous stop-trial. Interestingly, this was independent from whether the inhibition response in the previous stop-trial was successful or not. This pattern of results is an indication of the co-influence of inhibition processes on precision performance. It seems that participants strategically delayed their responses and waited for another stop-trial (Bisset & Logan, 2011; Verbruggen & Logan, 2017), at the expense of response precision. This is in line with previous studies using the SST that also reported a modulation of the current response (as signified by slower RTs) after a previous stop-trial (e.g., Rieger & Gauggel, 2010; Schachar et al., 2004; Verbruggen & Logan, 2008b; Verbruggen et al., 2008). However, the results were ambiguous with regard to response inhibition performance: While several studies (e.g., Verbruggen & Logan, 2008b; Schachar et al., 2004; Verbruggen et al., 2008) reported adjustments after unsuccessful response inhibition, which is reminiscent of post-error slowing, Rieger and Gauggel (2010) found response slowing after both, unsuccessful and successful stop-trials, which indicates a strategic delay of the current response after a stop-trial. Verbruggen et al. (2008)assume that prolonged responses after successful stop trials are due to repetition priming. In any case, at this point-in-time, we do not know if similar cognitive mechanisms are involved in the slowing of the RT in the SST and in the decrease of response precision in the present ARI task. The phenomenon itself, however, is interesting and the issue should gain more attention in sport psychological research.

Also, it should be noted that the participants in the present study were novices, that is, non-basketball players. This is a limitation, although one aim of the two experiments was to validate suitable (that is, dynamic sport-specific) stimulus material for constructing a basketball-specific ARI task. This was warranted, because the ARI task has only been used once in a sport-specific context before (in baseball; Gray, 2009), but with a different paradigm. To this end, basketball expertise was not required because at this point-in-time, we were not interested in expertise-novice differences, but to develop and validate the basketball-specific paradigm as such. However, the question if athletic expertise benefits response inhibition or not is certainly most relevant for research in sport psychology. Previous research using the SST demonstrated superior expert performance (e.g., Simonet et al., 2023; Albaladejo-Garcia et al., 2023; Fleddermann et al., 2023; Heppe & Zentgraf, 2019). At the same time, as was mentioned in the beginning of the introduction, even the most capable basketball players in the world fall for the pump fake in 73% of the time during real games in the NBA (Meyer et al., 2022a). This means that these most professional elite athletes are not able to anticipate the deception and inhibit the inappropriate response to the pump fake. As this real-world observation demonstrates, response inhibition in the basketball-specific ARI task may not be influenced by expertise. This is at least true for the performance outcome of successful vs. unsuccessful inhibition in stop-trials as such, not looking at response precision in go-trials. Whether there are differences between expert basketball players and novices in response inhibition performance (as signified by the PNR) should be the focus of future investigations.

### Practical implications and applicability

What lessons can be learned from the two experiments for sports practice? The images associated with the PNR in both experiments show the last foot contact on the ground and thus, represent the attacker’s “last chance” to change the action. According to the basketball rules, the attacker is not allowed to land again on the ground with the ball in his/her hands after a dribbling and a jump. The so called “traveling” would be penalized by losing possession of the ball to the opposing team (NBA, n.d.). This implies that it is possible to inhibit the response until the last foot contact of the attacker on the ground. But as mentioned in the introduction, kinematic parameters are also used to detect deceptive actions (Loffing & Canal-Bruland, 2017). A full hip and leg extension is not effective for a pump fake, as the chances of action is limited (Meyer et al., 2022c), but it is beneficial for a jump shot. In real situations, the deceptive action must be stopped much earlier to still be able to execute an alternative action.

Due to the dependence of inhibition and precision, defensive players tend to delay their reaction in further deceptive actions after a deceptive action of the attacker, because the expectation of a deceptive action is higher, thus decreasing the success of a deceptive action. It may be that this also depends on the player and is not a general phenomenon. In this context, player-dependent means that the defender judges from experience whether it is likely that the attacker will perform a pump fake, which influences the probability that the defender will block the attacker or not. The improved discrimination between fake and non-fake actions after observing fake actions also confirms this practical advice at a perceptual level (Kunde et al., 2019). In practice, this entailed analyzing the tendency of the opponent to perform a pump fake in order to increase the probability that the defender will not fall for a pump fake (Güldenpenning et al., 2018). In addition, successful and unsuccessful response inhibition in a previous attempt appears to have at least short-term effects on response precision in the next attempt (Bissett & Logan, 2011). This result suggests that defensive players are more likely to inhibit their response in the presence of another deception after a pump fake, as they would possibly delay their response, because they suspect another pump fake. This would imply that it is useful for an attacker to limit the number of deceptive actions within short periods of time or even to avoid a pump-fake repetition to keep effectiveness high.

## Conclusion

Together, the results of the present two experiments provide that the given stimulus material is suitable for investigating response inhibition for the basketball pump fake. The decrease in inhibition performance in both experiments confirms that the closer the time of stopping was compared to the anticipated target, the lower the probability was to stop the planned action. Similar inhibitory processes for interrupted jump shots (Experiment 1) and pump fakes (Experiment 2) suggest a relatively stable inhibitory control ability for different anticipation-response-inhibition tasks with the same response. While anticipation performance benefits from practice, response inhibition does not. Also, successful and unsuccessful response inhibition appears to have at least short-term effects on response precision. To examine response inhibition ability in a basketball-specific scenario, a more realistic response scenario should be created by implementing a defensive blocking action into the task scenario, where participants perform a jumping action to block the shot. It could then be examined if the PNR occurs earlier than in the present study because of the higher demands on motor planning, which would be in accordance with the memory-drum theory by Henry and Rogers (1960). Further, the tendency of an attacker to generate pump fakes could be considered in subsequent studies, as could be the influence of expertise on the ability of inhibition performance and the impact of long-term training effects.

## Data Availability

All data and stimulus materials are available at the open science framework (OSF) and can be accessed at https://osf.io/q94ub/?view_only=efd09d9483f24194a1352c9c0c5d2104.

## Footnotes

1 Please note here, that each frame is presented for 16.7 ms, and we used the end of the presentation of the target picture as reference point.

2 The synchronization was modified, which led to differences in the registration of the keystroke in Experiment 2.
